# HMGB1-targeted anti-inflammatory rBox A gene therapy for viral airway infections and systemic inflammatory disease models

**DOI:** 10.1128/mbio.01819-26

**Published:** 2026-08-24

**Authors:** Kari Ann Shirey, Daniel Prantner, Lynda Coughlan, Chanhee Park, Lin Zou, Wei Chao, John Joseph, Jorge C. G. Blanco, Stefanie N. Vogel

**Affiliations:** 1 Department of Microbiology and Immunology, University of Maryland School of Medicine 12264, Baltimore, Maryland, USA; 2 Center for Vaccine Development and Global Health, University of Maryland School of Medicine 12264, Baltimore, Maryland, USA; 3 Center for Shock, Trauma and Anesthesiology Research, University of Maryland Baltimore School of Medicine 12264, Baltimore, Maryland, USA; 4 Sigmovir Biosystems, Inc., Rockville, Maryland, USA; Duke University School of Medicine, Durham, North Carolina, USA

**Keywords:** HMGB1, TLR4, MD-2, influenza, LPS, mice, cotton rats, adenovirus, Box A

## Abstract

High mobility group box 1 (HMGB1) is a host-derived proinflammatory molecule released during cell injury. Elevated extracellular HMGB1 levels have been associated with disease severity. HMGB1 contributes to host influenza responses by stimulating TLR4/MD-2 signaling, amplifying lung inflammation, and tissue damage via cytokine production and leukocyte infiltration. HMGB1 also plays a central role in other inflammatory diseases, including sepsis and liver toxicity, making it both a biomarker of disease severity and a potential therapeutic target. We previously reported development of a non-replicating adenovirus (AdV) vector that carries an inflammation-inducible cassette that remains dormant in the absence of inflammation but produces recombinant HMGB1 Box A, a competitive antagonist of HMGB1, in response to inflammatory stimuli. Therapeutic treatment with AdV.C3-Tat/HIV-Box A mitigated lung and systemic inflammation in response to influenza in mice and cotton rats. Herein, we have optimized vector delivery and expanded its use in multiple models of infectious and non-infectious HMGB1-induced inflammation.

**IMPORTANCE:**

Despite promising preclinical outcomes, clinical translation of high mobility group box 1 (HMGB1) or TLR4/MD-2 antagonists for inflammatory diseases has been limited by challenges of drug synthesis, dosing, delivery, and off-target effects. Recombinant Box A (rBox A) is a competitive inhibitor of HMGB1 that binds to TLR4 and disrupts HMGB1-mediated TLR4/MD-2 signaling. rBox A exhibits broad anti-inflammatory activity across multiple diseases (e.g., endotoxemia, sepsis, neuroimmune disorders, ischemia/reperfusion); however, its therapeutic utility is constrained by the need for high, repeated dosing. We have engineered a non-replicating adenoviral (AdV) vector (AdV.C3-Tat/HIV-Box A) that produces recombinant (r)Box A when “sensed” by an inflammation-responsive complement C3 (C3) promoter. Therapeutic administration of AdV.C3-Tat/HIV-Box A resulted in significant protection and reduced inflammation in multiple HMGB1-mediated disease models, advancing a first-in-class, broadly applicable, host-directed gene therapy that selectively disrupts HMGB1-TLR4 signaling and has potential as both a broad-spectrum therapeutic for HMGB1-driven inflammatory diseases and a rapid-response countermeasure against emerging respiratory pathogens.

## INTRODUCTION

Influenza is a highly contagious respiratory illness. The WHO estimates that influenza-associated deaths globally range from ~300,000 to 650,000 (1), and the Centers for Disease Control and Prevention indicate that the 2024–2025 flu season was significantly more severe than initially projected, with 51 million illnesses, 23 million medical visits, 710,000 hospitalizations, and 45,000 deaths in the United States (2). The need to predict which major strains to include in each upcoming year’s vaccine, as well as the emergence of strains to which we have no prior exposure, has led to an ongoing effort to develop a “universal influenza vaccine” (3, 4). Although antiviral agents are available, their efficacy is limited by the need for early administration after infection (5) and by influenza’s ability to mutate and develop drug resistance (6, 7). Influenza-induced disease is initiated by viral replication that damages airway epithelial cells. The severe inflammatory response that follows, driven by metabolic stress in innate immune cells (e.g., macrophages [Mϕ]), ultimately elicits a “cytokine storm” that may lead to acute lung injury (ALI) or the more severe respiratory distress syndrome (8, 9).

High mobility group box 1 (HMGB1), a redox-sensitive nuclear protein that is actively secreted or released during cellular injury (10), is widely regarded as a key driver of infection-induced and sterile inflammation because it functions as a “damage-associated molecular pattern” (DAMP). HMGB1 activates multiple innate immune receptors, including TLR2, TLR4, TLR9, and RAGE, thereby promoting cytokine production, leukocyte recruitment, and tissue injury (10–15). In this way, extracellular HMGB1 is not just a biomarker of tissue injury; it initiates, sustains, and amplifies inflammation through a positive feedback loop that increases cytokine production and immune-cell activation. Studies also show that the disulfide form of HMGB1 is especially important for signaling by TLR4 and its co-receptor MD-2, further supporting its role in hyperinflammatory states (10). Using TLR4-deficient mice and the TLR4/MD-2 antagonist, Eritoran, we previously reported that influenza-induced lung inflammation and lethality are mediated by TLR4/MD-2 (16); however, because the influenza virus does not express “pathogen-associated molecular patterns” (PAMPs) that activate TLR4/MD-2, this led to the identification of host-derived HMGB1 as the key driver of influenza-mediated disease, as evidenced by the efficacy of a small molecule HMGB1 antagonist, P57799, to block disease equivalently to Eritoran (17, 18).

HMGB1 contains two DNA-binding domains, Box A and Box B (19). Using surface plasmon resonance, He et al. (20) demonstrated that the Box A domain of full-length HMGB1 binds to TLR4, thereby facilitating the binding of Box B to MD-2. Recombinant Box A (rBox A), which is identical to the Box A sequence in HMGB1, competitively inhibits full-length HMGB1 binding to TLR4, thereby blocking TLR4/MD-2-mediated signaling (diagrammed in our previous report [21]). Anti-HMGB1 antibodies, small-molecule inhibitors, rBox A, and other structurally unrelated antagonists reduce inflammation and organ damage in models of inflammation, including endotoxicity, sepsis, ischemia-reperfusion injury, hemorrhagic shock, trauma, and collagen-induced arthritis (22–29). Together, these studies establish HMGB1 as a conserved host-derived molecule that links tissue damage to innate immune activation across diverse infectious and non-infectious inflammatory diseases, making it a compelling therapeutic target. However, to date, no HMGB1 antagonist has reached the clinic due to constraints related to delivery, synthesis, dosing, and off-target effects, with inflammation being only partially ameliorated (16, 30–32).

To circumvent these problems, we engineered an inflammation-inducible rBox A expression cassette delivered by a non-replicating adenoviral vector, “AdV.C3-Tat/HIV-Box A,” that significantly mitigates lung and systemic inflammation when administered therapeutically to influenza-infected mice and cotton rats (*Sigmodon hispidus*; CR) (21). Herein, we optimized our gene therapy for influenza treatment and have extended its therapeutic use to other infectious and non-infectious models in which HMGB1-mediated TLR4/MD-2 signaling is the central driver of disease-induced inflammation.

## RESULTS

### Optimization of AdV.C3-Tat/HIV-Box A treatment for influenza infection of mice

Our previous study evaluated three AdV vectors: AdV.C3-Tat/HIV-Luc (luciferase-producing control vector), AdV.C3-Tat/HIV-Box A^Ser^ (wild-type [WT] Box A sequence), and AdV.C3-Tat/HIV-Box A^Gly^ (mutated Box A sequence to prevent glycosylation). As diagrammed in our original report (21), the AdV vector encodes rBox A using a two-component expression system in which an inflammation-responsive mouse C3 promoter drives HIV *Tat* expression. Tat subsequently activates rBox A transcription via an LTR promoter within the non-replicating AdV vector (21). *In vitro*, LPS induced comparable levels of rBox A^Ser^ or rBox A^Gly^ in AdV-infected Mϕ, indicating that glycosylation did not affect secretion of rBox A (21). However, when mice were infected with a lethal dose of mouse-adapted A/PR/8/34 (PR8), and treated i.v. on day 1 post-infection (p.i.) with 2 × 10^7^ PFU of AdV.C3-Tat/HIV-Box A^Ser^, AdV.C3-Tat/HIV-Box A^Gly^, or an equal mix of the two Box A vectors (AdV.C3-Tat/HIV-Box A^Ser/Gly^), AdV.C3-Tat/HIV-Box A^Gly^ failed to protect the mice, whereas AdV.C3-Tat/HIV-Box A^Ser^ and AdV.C3-Tat/HIV-Box A^Ser/Gly^ elicited up to 60% survival. Increasing the AdV dose 10-fold to 2 × 10^8^ PFU/mouse increased protection to 60% by all three vectors. As expected, AdV.C3-Tat/HIV-Luc failed to protect the mice at either dose (21).

**Fig 1:**
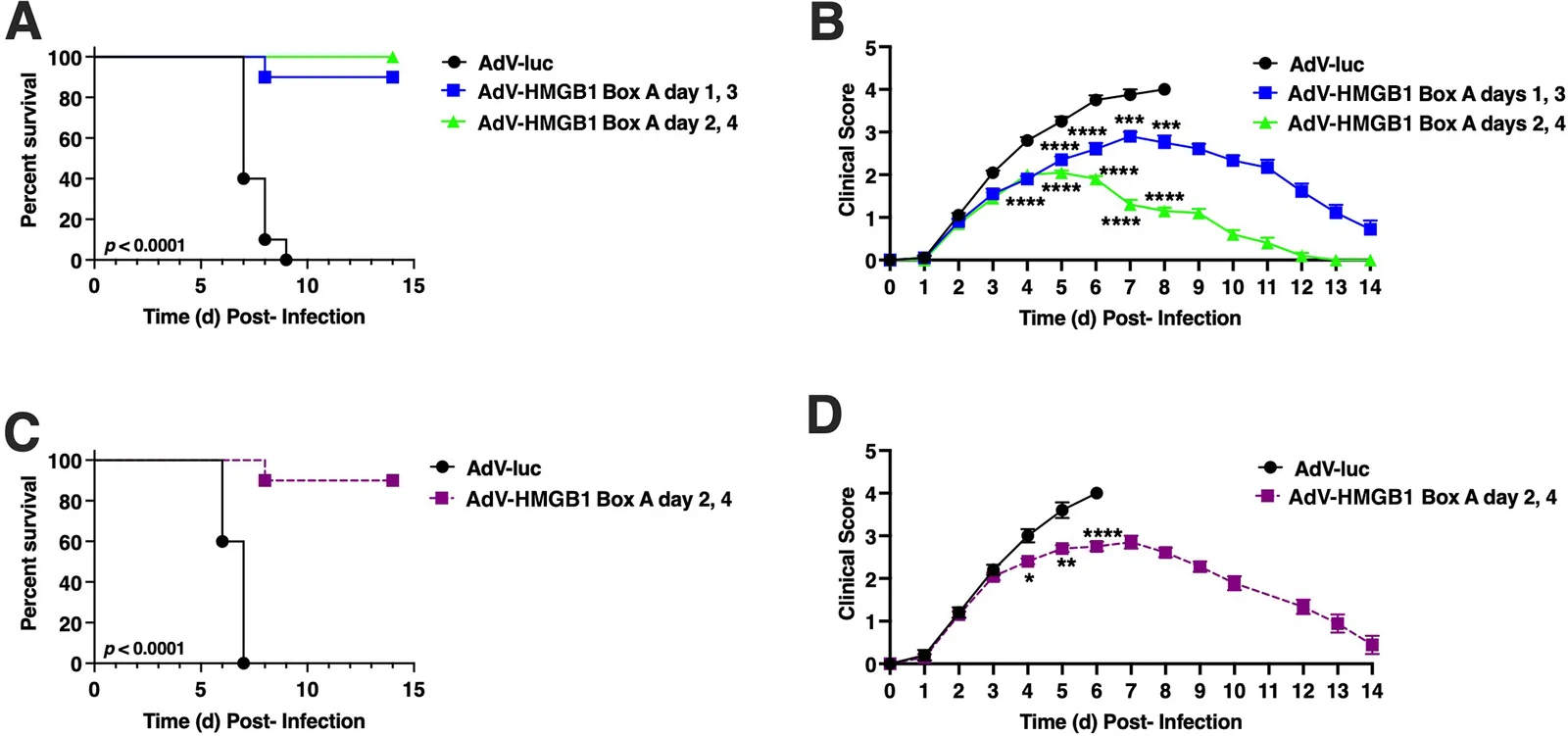
Optimization of AdV.C3-Tat/HIV-Box A. (**A, B**) C57BL/6J mice were infected on day 0 with PR8 (~7,500 TCID_50_). Mice were treated i.v. with AdV.C3-Tat/HIV-Luc (“AdV-luc”) or AdV.C3-Tat/HIV-Box A^Ser/Gly^ (“AdV-HMGB1 Box A”; 2 × 10^7^ PFU/mouse) on days 1 and 3 or AdV.C3-Tat/HIV-Box A^Ser/Gly^ (“AdV-HMGB1 Box A”; 2 × 10^8^ PFU/mouse) on days 2 and 4 p.i. Mice were monitored daily for survival (**A**) and clinical scoring (**B**). (**C, D**) C57BL/6J mice were infected on day 0 with PR8 (LD_90_). Mice were treated with AdV.C3-Tat/HIV-Luc (2 × 10^8^ PFU/mouse) or with AdV.C3-Tat/HIV-Box A^Ser^ (“AdV-HMGB1 Box A”; 2 × 10^8^ PFU/mouse) by i.v. injection and monitored for survival (**C**) and clinical scoring (**D**). Data are from two separate experiments with five mice/treatment/experiment. Survival data were analyzed by Mantel-Cox log-rank test. Statistics for clinical scores for AdV-HMGB1 Box A treatments compared with AdV-Luc were analyzed by one-way ANOVA. *P = 0.015; **P = 0.0054; ***P = 0.0001; ****P < 0.0001.

To further optimize treatment (21), we systematically modified vector dose and timing to optimize treatment for survival. When PR8-infected mice were treated with two doses of AdV.C3-Tat/HIV-Box A^Ser/Gly^ (2 × 10^7^ PFU/mouse) on days 1 and 3 p.i., survival increased to 90% with a corresponding decrease in clinical scores (Fig. 1A and B, blue). When the Ser/Gly variant mix (Fig. 1A, green) or the Ser variant only (Fig. 1C, purple) (2 × 10^8^ PFU/mouse) was administered on days 2 and 4 p.i., 100% and 90% survival, respectively, were achieved, which also correlated with decreased clinical scores (Fig. 1B and D, green and purple, respectively). This same level of protection is comparable to that achieved by daily high-dose administration of either Eritoran or P5779 from days 2 to 6 p.i. (16, 17). Delaying treatment until days 3 and 4 p.i. reduced survival to 60% (6/10 mice survived). Since AdV.C3-Tat/HIV-Box A^Ser/Gly^ and AdV.C3-Tat/HIV-Box A^Ser^ were comparably effective when given either i.v. or i.p. (data not shown), we used these interchangeably for subsequent *in vivo* experiments.

**Fig 2:**
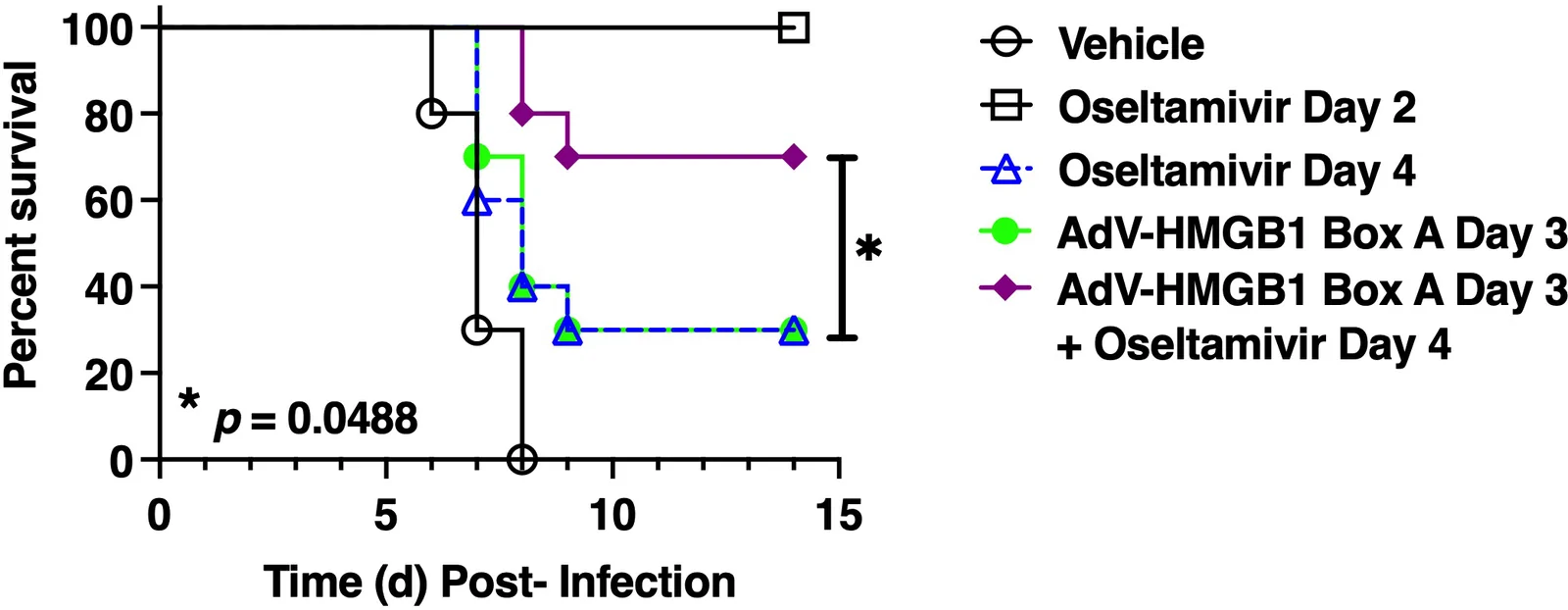
Effect of combined treatment of PR8-infected mice with oseltamivir and AdV.C3-Tat/HIV-Box A. C57BL/6J mice were infected with mouse-adapted influenza strain PR8 (~7,500 TCID_50_). Vehicle or oseltamivir (1 mg/mouse, p.o.) only was administered on days 2 - 6 or 4 - 6 p.i. AdV.C3-Tat/HIV-Box A^Ser/Gly^ (“AdV-HMGB1 Box A”; 2 × 10^8^ PFU/mouse, i.v.) was administered i.v. on day 3 p.i. Responses to the individual or combined treatments are shape- and color-coded in the graph as follows: vehicle (black open circle), oseltamivir, day 2 p.i. (open black square), oseltamivir, day 4 (blue, open triangle), AdV.C3-Tat/HIV-Box A^Ser/Gly^ (green circle), or both AdV.C3-Tat/HIV-Box A^Ser/Gly^ and oseltamivir (day 4; purple diamond). Survival was monitored daily. The data represent the combined results of two separate experiments (*N* = 5 mice/treatment/experiment). Data were analyzed by Mantel-Cox log-rank test.

We next sought to determine the efficacy of AdV.C3-Tat/HIV-Box A administered alone or in combination with the neuraminidase inhibitor oseltamivir. Generally, oseltamivir must be given within 2 days of symptom onset to be effective (33, 34). We compared the survival of mice lethally infected with PR8 on day 0 and treated with vehicle (saline), oseltamivir alone (administered either on days 2–6 or days 4–6 p.i.), or AdV.C3-Tat/HIV-Box A on day 3 p.i. alone or in combination with oseltamivir. We chose these time points as our previous studies with oseltamivir administered at day 4 p.i. or AdV.C3-Tat/HIV-Box A on day 3 p.i. showed comparably reduced protection after the onset of symptoms (17, 21). When oseltamivir was administered starting on day 2 p.i., highly significant protection was observed (Fig. 2, open square), but survival decreased from 100% to ~30% when oseltamivir treatment was delayed until day 4 p.i. (Fig. 2, open blue triangle). Treatment with AdV.C3-Tat/HIV-Box A alone on day 3 p.i. also resulted in ~30% protection (Fig. 2, green). However, when mice were treated with AdV.C3-Tat/HIV-Box A on day 3 and oseltamivir on day 4, a significant increase in survival was observed (Fig. 2, purple). Thus, combined therapy with oseltamivir and AdV.C3-Tat/HIV-Box A extends the therapeutic window and provides protection beyond that achieved with either alone.

**Fig 3:**
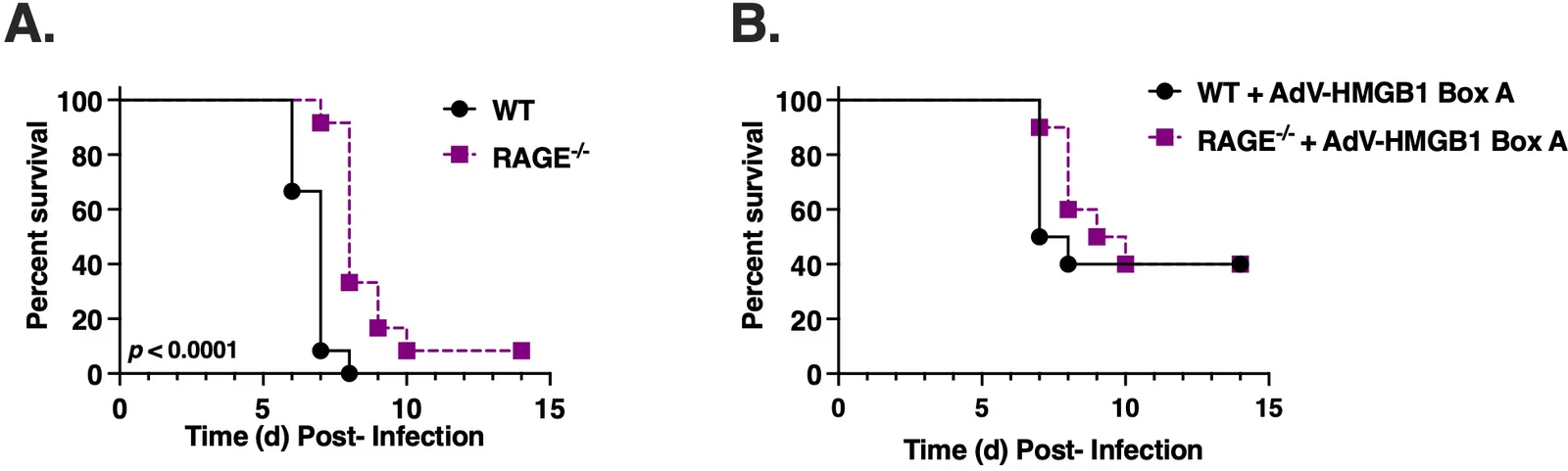
AdV.C3-Tat/HIV-Box A treatment in RAGE^−/−^ mice protects against lethal influenza infection. (**A**) Susceptibility of RAGE^−/−^ mice to influenza PR8 infection. WT (black circles) and RAGE^−/−^ (purple squares) mice were infected with influenza PR8 (~7,500 TCID50; i.n.) and monitored daily for survival for 14 days. WT, *N* = 11; RAGE^−/−^, *N* = 11. (**B**) C57BL/6J and RAGE^−/−^ mice were infected on day 0 with PR8 (LD_90_) and treated once i.v. with AdV.C3-Tat/HIV-Box A^Ser/Gly^ (“AdV-HMGB1 Box A”; 2 × 10^7^ PFU/mouse) 24 h after PR8 infection. Mice were monitored for survival for 14 days. Data are from two separate experiments with five mice/treatment/experiment. Data were analyzed by Mantel-Cox log-rank test.

HMGB1 binds to and activates both TLR4/MD-2 and RAGE. PR8-infected RAGE^−/−^ mice are comparably susceptible to WT mice, with a slight extension of the mean time to death (Fig. 3A) (21, 35), in contrast to the refractory response observed in PR8-infected TLR4^−/−^ mice (16, 36). AdV.C3-Tat/HIV-Box A therapy on day 1 p.i. improved survival comparably in both WT and RAGE^−/−^ mice (~60% survival), yet RAGE^−/−^ mice still exhibited a slight extension of the mean time to death (Fig. 3B). These data support the conclusion that rBox A primarily targets TLR4/MD-2-mediated signaling in response to PR8 infection.

### Efficacy of AdV.C3-Tat/HIV-Box A on respiratory syncytial virus-induced inflammation in CR

**Fig 4:**
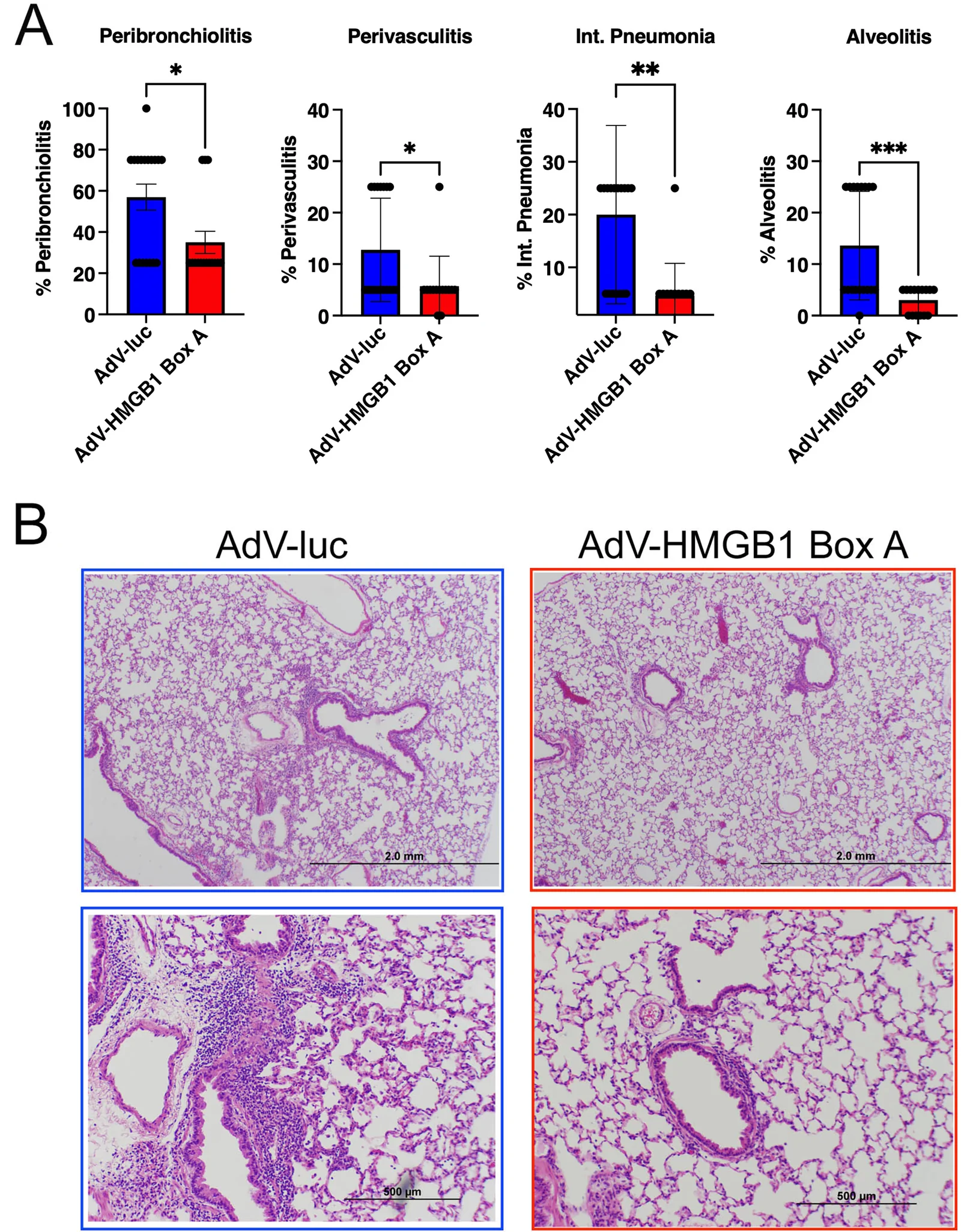
AdV.C3-Tat/HIV-Box A treatment reduces RSV-induced lung inflammation in CR. CR (*n* = 6/group/experiment; two separate experiments) were challenged with 100 μL i.n. of RSV A2 at a dose of 5 × 10^5^ PFU/CR. CR in each group were treated i.p. on days 1 and 3 with AdV.C3-Tat/HIV-Luc (“AdV-luc”) or AdV.C3-Tat/HIV-Box A^Ser^ (“AdV-HMGB1 Box A”; 5 × 10^8^ PFU/CR). All CR were sacrificed on day 5 p.i., and lungs were harvested, fixed, and hematoxylin and eosin (H&E) stained for quantification of histopathology. (**A**) Results of blinded scoring of H&E-stained lung sections comparing CR treated with AdV.C3-Tat/HIV-Box A^Ser^ (red bars) vs CR treated with the AdV.C3-Tat/HIV-Luc control vector (blue bars), where a significant reduction of all four lung infiltration histological parameters was recorded in AdV.C3-Tat/HIV-Box A^Ser^-treated CR. (**B**) Representative microphotographs of the reduced peribronchiolitis and perivasculitis (top, scale bars, 2 mm)*,* and perivasculitis, interstitial pneumonia, and alveolitis (bottom, scale bars, 500 μm) achieved by treatment of CR with AdV.C3-Tat/HIV-Box A^Ser^.

Respiratory syncytial virus (RSV) is a globally significant cause of lower respiratory tract infections in the very young and the elderly (37–40). RSV infection induces significant levels of HMGB1 (18), and more recently, the RSV nonstructural protein NS1 was shown to drive HMGB1 release through direct interaction with histone H1.0, triggering the active release of HMGB1 from the nucleus into the cytoplasm and extracellular space (41). To assess the efficacy of AdV.C3-Tat/HIV-Box A in reducing RSV-induced lung inflammation, CR were infected with RSV and then treated with AdV.C3-Tat/HIV-Box A^Ser^ on days 1 and 3 post-infection. CR were euthanized, and lungs were harvested for histopathology on day 5 p.i. Treatment with AdV.C3-Tat/HIV-Box A^Ser^ (red bars) significantly reduced lung pathology across all four quantified parameters (peribronchiolitis, perivasculitis, interstitial pneumonia, and alveolitis) compared to CR treated with the control vector, AdV.C3-Tat/HIV-Luc (blue bars) (Fig. 4A). Representative micrographs (Fig. 4B; top panels; low power resolution; Fig. 4B; bottom panels; high power resolution) show the significant reduction in lung inflammation achieved by treatment of CR with AdV.C3-Tat/HIV-Box A^Ser^.

### Efficacy of AdV.C3-Tat/HIV-Box A treatment on additional models of inflammation

**Fig 5:**
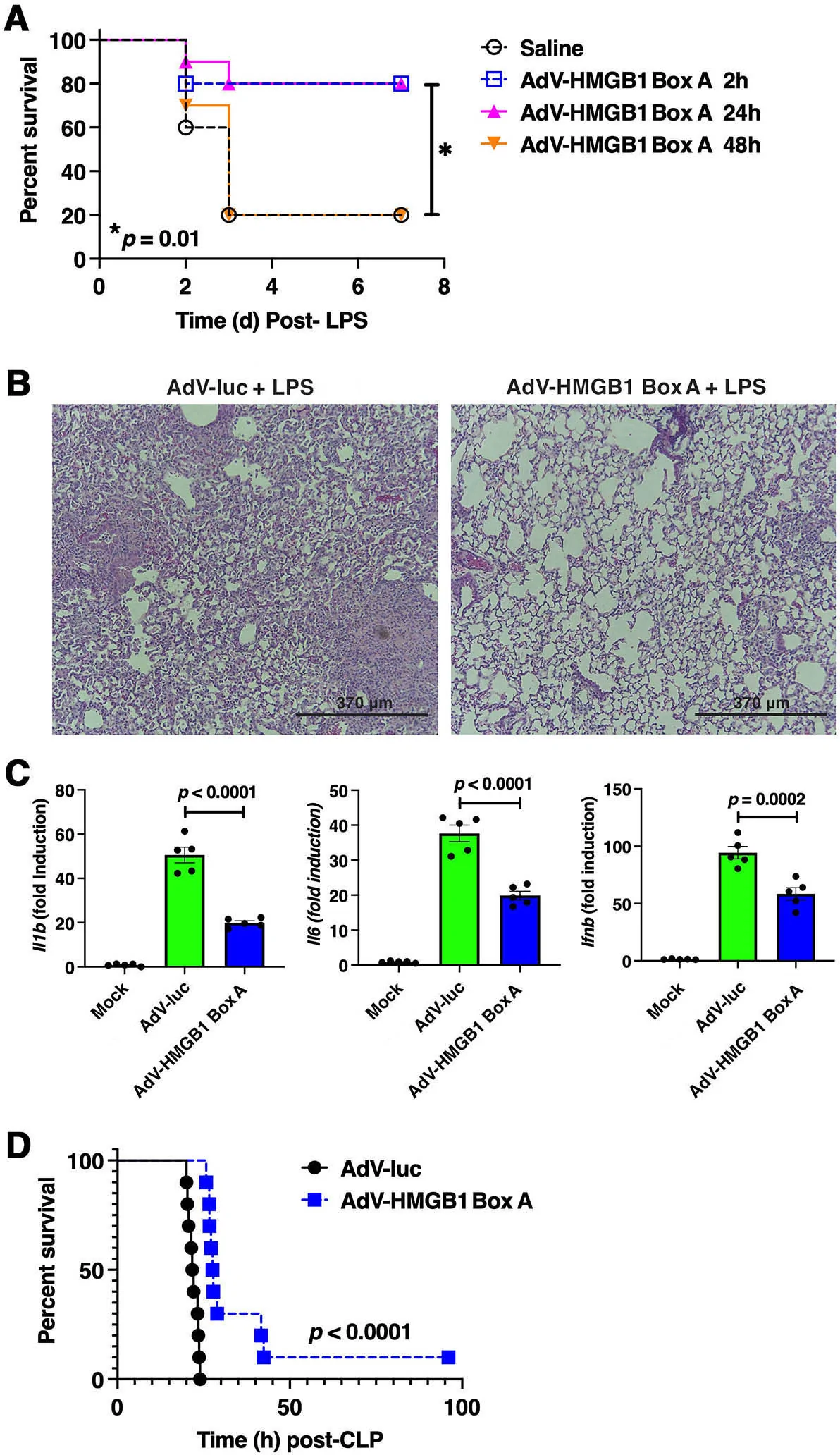
AdV-HMGB1 Box A treatment reduces inflammatory response in LPS and sepsis models of inflammation. (**A**) C57BL/6J mice were injected with LPS (300 μg/mouse, i.p.) and then treated i.v. with AdV.C3-Tat/HIV-Box A^Ser/Gly^ (“AdV-HMGB1 Box A”; 2 × 10^8^ PFU/mouse) at 2, 24, or 48 h later. Data are combined from two separate experiments (*N* = 5/treatment/experiment) except for the 2 h treatment that was carried out only once (*N* = 5/treatment). Data were analyzed by Mantel-Cox log-rank test. (**B and C**) C57BL/6J mice were administered LPS (20 μg/mouse; intratracheal [i.t.]) and were treated with AdV.C3-Tat/HIV-Box A^Ser^ (“AdV-HMGB1 Box A”; 2 × 10^8^ PFU/mouse; i.v.) 24 h later. Lungs were harvested 3 days later for (**B**) histopathology and (**C**) gene expression measurements. *N* = 5/group. Analysis was carried out by one-way ANOVA. (**D**) Male C57BL/6J mice were subjected to cecal ligation puncture (CLP) and treated i.p. with AdV.C3-Tat/HIV-Luc (“AdV-luc”) or AdV.C3-Tat/HIV-Box A^Ser/Gly^ (“AdV-HMGB1 Box A”; 2 × 10^8^ PFU/mouse) 2 h post-surgery. Mice were monitored for survival for 7 days. Data are derived from two separate experiments with five mice/treatment/experiment. Data were analyzed by Mantel-Cox log-rank test.

Wang et al. originally reported that LPS-induced lethality was HMGB1-mediated (42). To determine if AdV.C3-Tat/HIV-Box A would protect against other severe inflammatory responses, mice were LPS-challenged (300 μg/mouse i.p.) and treated i.v. with AdV.C3-Tat/HIV-Box A^Ser/Gly^ at 2, 24, or 48 h later. The kinetics of lethality to LPS are considerably more rapid than observed with PR8 infection (16, 17, 21); nonetheless, treating the mice at 2 or 24 h post-LPS led to highly significant survival (Fig. 5A). Our data also show that delaying treatment until 48 h after LPS challenge fails to protect the mice, indicating that the HMGB1-TLR4 signaling must occur within the first 2 days after systemic LPS challenge.

Intratracheal (i.t.) LPS is a common model of ALI. Mice were administered AdV.C3-Tat/HIV-Luc or AdV.C3-Tat/HIV-Box A^Ser/Gly^ (2 × 10^8^ PFU/mouse) 24 h after i.t. LPS administration (25 μg/mouse) and were euthanized 3 days later to assess the lung inflammatory response. Treatment with AdV.C3-Tat/HIV-Box A^Ser/Gly^ reduced lung pathology (Fig. 5B) and was accompanied by decreased lung *Il1b*, *Il6*, and *Ifnb* gene expression (Fig. 5C).

While LPS administration provides a tractable model of acute, TLR4-driven, HMGB1-mediated inflammation, it lacks the complexity and temporal urgency of clinically relevant polymicrobial sepsis. Cecal ligation puncture (CLP) is a well-characterized model that elicits rapid HMGB1 release due to polymicrobial infection, providing a clinically relevant platform with which to evaluate the efficacy of HMGB1-targeted interventions under conditions where treatment must be initiated after disease onset (43). A single i.p. dose of AdV.C3-Tat/HIV-Box A^Ser/Gly^ administered 2 h after CLP resulted in a statistically significant extension of the mean time to death, with a slight improvement in survival compared to mice treated with AdV.C3-Tat/HIV-Luc (Fig. 5D).

### Effect of AdV vectors on liver function

The AdV doses used in optimization studies are considerably lower than those typically used in vaccines or gene therapy (10^10^ to 10^11^) (44). To ensure that the Box A vector would not induce liver toxicity, hepatic enzymes, alanine transaminase (ALT) and aspartate transaminase (AST), were measured in serum 24 h after i.v. challenge of mice with 2 × 10^8^ or 2 × 10^9^ PFU AdV.C3-Tat/HIV-Luc. A dose of 2 × 10^8^ PFU/mouse failed to cause significant elevation of either ALT or AST compared to mock-treated mice (Fig. S1, top panels). Although the higher dose of 2 × 10^9^ PFU caused a significant increase compared to mock-treated mice, this level falls within the average range for untreated mice (<32 U/L for ALT and <60 U/L for AST) (45). To confirm these findings in a second rodent model, we utilized CR that are uniquely susceptible to non-adapted human respiratory viruses (46–48). CR were pre-bled to establish baseline ALT and AST levels and were then injected with low (1 × 10^8^ PFU) and high (1 × 10^9^ PFU) doses of AdV.C3-Tat/HIV-Box A^Ser^. Serum was collected 2 and 24 h later. CR were then injected with LPS i.p., and serum was collected 24 h later. Similar to our observations in mice, AST levels increased only with the higher dose (1 × 10^9^ PFU) at 24 h after AdV treatment (Fig. S1, bottom right panel). While treatment increased ALT levels, even with a low dose of vector (1 × 10^8^ PFU), both ALT and AST levels remained within the normal range for CR (<220 U/L for ALT and <200 U/L for AST) (49, 50). Thus, the minimal effect of the vector on enzymes associated with liver toxicity, even at a 10-fold higher AdV dose than shown to protect mice and CR from influenza, further supports the safety of our vectors, consistent with a lack of lung inflammation at high doses as we reported previously (21).

**Fig 6:**
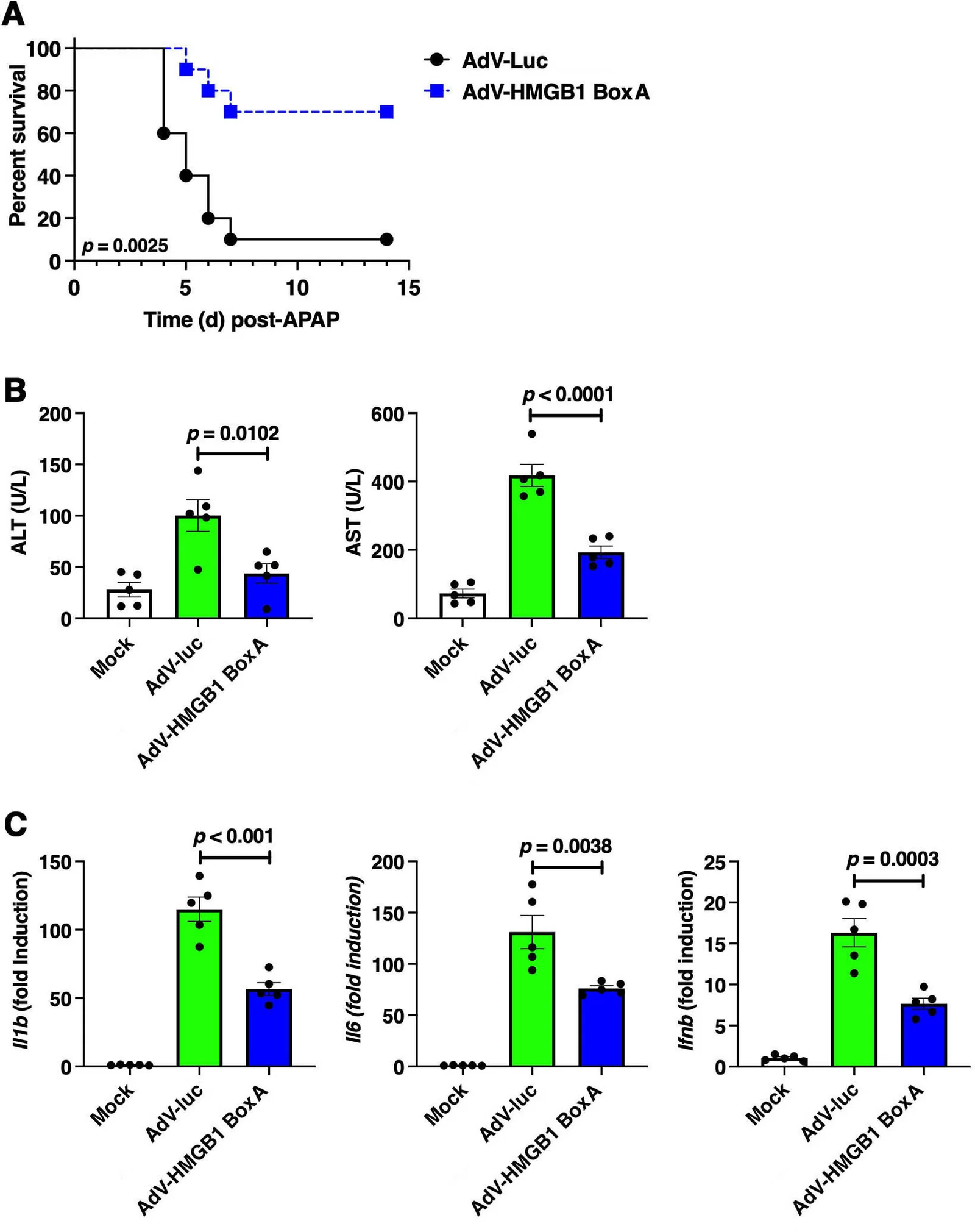
AdV.C3-Tat/HIV-Box A protects against APAP-induced liver injury. Male C57BL/6J mice were fasted overnight and received APAP (350 mg/kg) by i.p. injection. On days 1 and 3 post-APAP, mice received AdV.C3-Tat/HIV-Luc (“AdV-Luc”) or AdV.C3-Tat/HIV-Box A^Ser^ (“AdV-HMGB1 Box A”; 2 × 10^8^ PFU/mouse) i.p. Mice were monitored for (**A**) survival for 14 days post-APAP or (**B and C**) harvest studies. For the harvest studies, on day 5 post-APAP, serum was collected for AST and ALT analysis by ELISA. Mice were then euthanized and the livers harvested for gene expression analysis. *N* = 5 mice/group. (**A**) Data were analyzed by Mantel-Cox log-rank test. (**B and C**) Data were analyzed by one-way ANOVA with Tukey’s post hoc test.

HMGB1 was reported to play an important role in acetaminophen (APAP)-induced liver toxicity that leads to death in mice (43, 51, 52). We next evaluated if treatment with AdV.C3-Tat/HIV-Box A would be protective in this model. Mice were injected with APAP (350 mg/kg) and administered AdV.C3-Tat/HIV-Luc or AdV.C3-Tat/HIV-Box A^Ser^ on days 1 and 3 post-APAP. Survival was monitored for 14 days. Mice that received AdV.C3-Tat/HIV-Luc exhibited 90% lethality, in contrast to 70% survival in mice treated with AdV.C3-Tat/HIV-Box A^Ser^ (Fig. 6A). To extend these findings, mice were injected with APAP and treated with AdV.C3-Tat/HIV-Luc or AdV.C3-Tat/HIV-Box A^Ser^ as described above. On day 5 post-APAP, serum was collected for AST and ALT analysis, and liver mRNA was prepared for gene expression analysis. AdV.C3-Tat/HIV-Box A^Ser^ treatment significantly reduced APAP-induced elevations in AST and ALT (Fig. 6B) and proinflammatory cytokine mRNA (*Il1b*, *Il6*, *Ifnb*; Fig. 6C).

### Effect of AdV.C3-Tat/HIV-Box A on LPS-treated human HL-60 Mϕ

**Fig 7:**
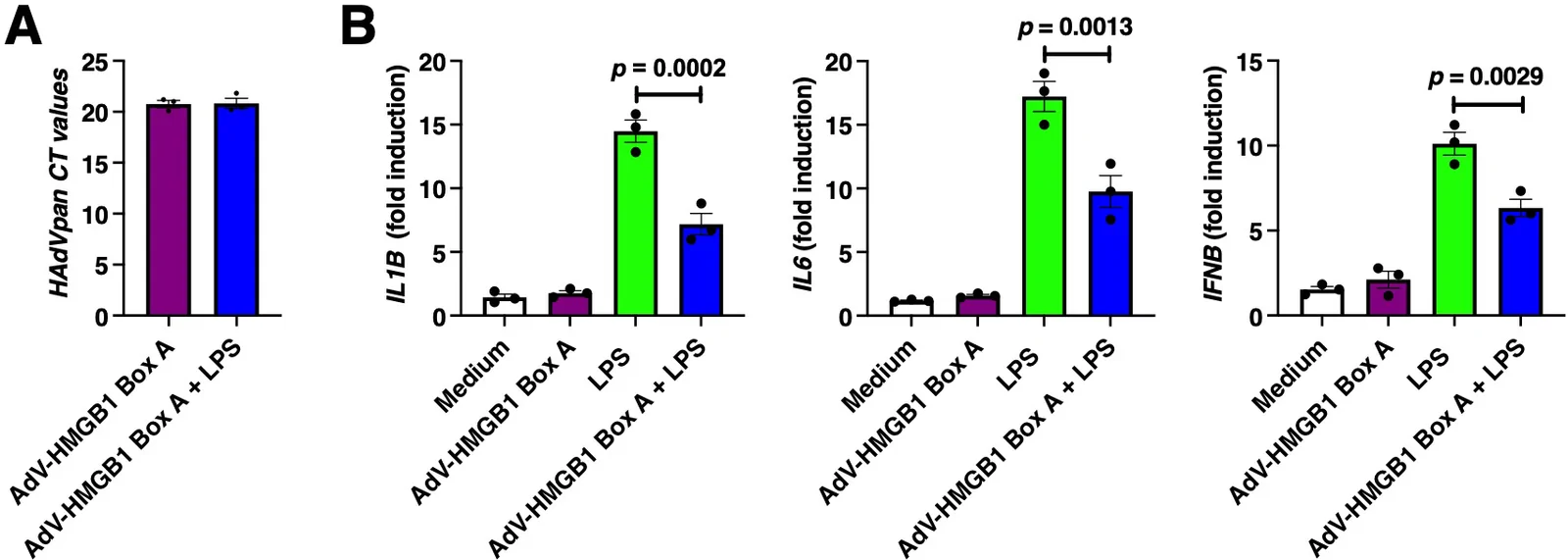
Human HL-60 cells infected with AdV-HMGB1 Box A. Differentiated HL-60 Mϕ were infected with AdV.C3-Tat/HIV-Box A^Gly^ (“AdV-HMGB1 Box A,” MOI = 2) for 24 h. After 24 h, cells were stimulated with LPS (10 ng/mL) for 20 h. RNA was isolated and gene expression analyzed for hAdVpan (**A**) and cytokines (**B**). Data shown are combined from three separate experiments. Data were analyzed by one-way ANOVA.

To date, our studies have been conducted in rodents (mice and CR) or in their Mϕ (21). The human monocytic cell line, HL-60, was treated with phorbol myristic acetate (PMA) to differentiate into Mϕ, then infected with AdV.C3-Tat/HIV-Luc or AdV.C3-Tat/HIV-Box A for 24 h. Mϕ cultures were then treated with LPS (10 ng/mL) for an additional 20 h. Figure 7A shows that the cells were equally infected, as evidenced by comparable levels of human AdV (HAdV) RNA. Cytokine gene expression (human *IL1B*, *TNF*, *IFNB*) was upregulated in LPS-treated cells but reduced in cells treated with the Box A-producing vector. These findings indicate that AdV.C3-Tat/HIV-Box A also blunts LPS-induced cytokine gene expression in human Mϕ.

## DISCUSSION

There is strong experimental support for HMGB1 as a mediator of inflammation in both infectious and non-infectious disease models, owing to its ability to activate innate receptors, including RAGE, TLR4/MD-2, TLR2, and TLR9 (53). Yang et al. first demonstrated that the disulfide HMGB1 isoform binds directly to MD-2. MD-2 deficiency or silencing abrogates HMGB1-dependent TLR4 signaling in Mϕ, and HMGB1-driven inflammatory injury is ameliorated by P5779, a peptide antagonist that selectively binds MD-2, thereby blocking the HMGB1–TLR4/MD-2 signaling axis while sparing LPS responses (43). HMGB1 instillation into the lung induces leukocyte infiltration and inflammatory cytokine production through TLR4-dependent signaling (54). This is important because it establishes that HMGB1 uses the same receptor complex architecture as the canonical TLR4 ligand, LPS, with MD-2 conferring ligand recognition specificity.

In both infectious and sterile inflammation models, this HMGB1-mediated inflammation has been linked to hepatic ischemia-reperfusion injury, chemical toxicity, and other damage-associated settings in which HMGB1 release precedes Mϕ activation, cytokine amplification, and organ injury (12, 13). Consistent with this mechanism, multiple studies of HMGB1-driven inflammation report that anti-HMGB1 antibodies (e.g., m2G7) and selective inhibitors, such as P5779, suppress HMGB1/TLR4-mediated inflammatory responses without broadly suppressing PAMP-driven antimicrobial signaling (43, 55, 56), underscoring a therapeutically tractable separation between sterile DAMP sensing and host-defense TLR4 activity. Optimization of dosing and timing markedly improved efficacy against murine influenza infection. Specifically, vector administration on days 1 and 3, or on days 2 and 4, p.i. resulted in 90%–100% survival (Fig. 1), whereas administration on day 1 only, day 3 only, or days 3 and 4 were less protective (21; data not shown). This is consistent with the possibility that the AdV vector is likely being cleared or inactivated after a relatively short period of time or that transcription and translation of rBox A from the vector are sustained for no more than 1–2 days. That comparable protection was observed when either the mix of serine/glycine variants or the AdV.C3-Tat/HIV-Box A^Ser^ vectors were administered indicates that the WT (Ser) vector is sufficient.

Combination therapy for influenza and other respiratory infections is increasingly framed as pairing a pathogen-directed antiviral with a host-directed therapy to impair viral replication while mitigating harmful inflammatory host-response pathways. For influenza, this strategy is attractive because the virus rapidly mutates, whereas host factors are less variable, and host-directed approaches may therefore reduce resistance pressure and broaden activity against divergent strains. We previously reported that blocking host inflammatory signaling alone improved survival: in mouse studies, the TLR4 antagonist Eritoran or the HMGB1 antagonist P5779 reduced influenza-induced cytokine production, lung inflammation, and lethality (16, 17); however, in conjunction with delayed administration of oseltamivir, delayed targeting of the HMGB1-driven TLR4/MD-2 signaling pathway with Eritoran not only complemented antiviral therapy but also extended the therapeutic window (17). This combined approach is important because in severe respiratory infections, especially influenza pneumonia, pathology is driven not only by viral burden but also by excessive epithelial injury, cytokine response, and dysregulated innate immunity, so a purely antiviral drug may not fully address disease severity once inflammation is established. Here, we report that the combined use of AdV.C3-Tat/HIV-Box A with oseltamivir significantly increased survival in mice lethally infected with influenza (Fig. 2). Our findings are significant because the treatments were delayed such that oseltamivir alone was significantly less protective and the mice already showed clinical symptoms (Fig. 1), consistent with when individuals typically seek medical attention (57, 58). Other preclinical and translational studies show that combined anti-viral/immunomodulator regimens improve virologic suppression and lung protection compared with monotherapy, while allowing lower doses of each treatment and thereby potentially reducing toxicity (59, 60), with the central goal of preserving antiviral defense while limiting immunopathology (16, 17, 21, 59–61).

Both HMGB1–RAGE and HMGB1–TLR4 interactions mediate damage-associated inflammatory signaling but engage partially distinct downstream pathways, resulting in differential modulation of immune responses. HMGB1 binding to RAGE is commonly described as an amplification axis that promotes sustained NF-κB and MAPK activation, upregulates RAGE expression, and increases TLR4 surface availability, thereby establishing a feed-forward loop that prolongs inflammation (62). In addition, HMGB1–LPS complexes can be internalized via RAGE-mediated endocytosis, leading to lysosomal destabilization and the release of LPS into the cytosol, where it activates caspase-4/11 and drives noncanonical inflammasome signaling (15). In contrast, HMGB1 binding to the TLR4/MD-2 complex activates both MyD88-dependent and TRIF-dependent pathways, resulting in NF-κB and IRF-3 activation and robust production of pro-inflammatory cytokines (62, 63). Consistent with these mechanistic differences, we and others have shown that RAGE-deficient (RAGE^−/−^) mice succumb to PR8 influenza infection, suggesting that HMGB1 signaling in this context is predominantly mediated through TLR4/MD-2 (21, 35). Furthermore, treatment of PR8-infected RAGE^−/−^ mice with AdV.C3-Tat/HIV-Box A improved survival to levels comparable to WT controls (Fig. 3), providing additional support that influenza-induced HMGB1 signaling proceeds primarily through the TLR4/MD-2 axis. Moreover, P5779, which protects mice from lethal PR8 infection (17), does not inhibit RAGE-mediated endocytosis of HMGB1 or HMGB1/LPS complexes (22, 42, 43), further supporting the key role of HMGB1-mediated TLR4/MD-2 stimulation in influenza-induced disease.

The efficacy of AdV.C3-Tat/HIV-Box A in mice and in CR across diverse infectious and non-infectious inflammatory models, including RSV infection, endotoxicity, LPS-induced ALI, and APAP-induced liver toxicity, demonstrates its broad therapeutic potential beyond our initial influenza studies. We additionally evaluated our vector in a more severe CLP model (dual puncture without antibiotic intervention), which drives accelerated disease progression. This phenotype may reflect a shift in the temporal dynamics and magnitude of HMGB1 release following CLP, potentially exceeding the neutralizing capacity of rBox A. Alternatively, severe infection may impair vector-mediated rBox A expression or delay its induction relative to the early surge in HMGB1. It is also plausible that parallel HMGB1-independent inflammatory pathways are engaged under these conditions, thereby limiting the protective efficacy of the vector. Although additional optimization will be necessary to maximize efficacy in these distinct disease contexts, our findings provide strong proof-of-concept for the AdV.C3-Tat/HIV-Box A vector platform as a versatile strategy for future preclinical development.

Adenoviral vectors effectively infect many cell types, making them useful for efficient *in vivo* delivery (44, 64). They can produce robust, rapid expression of the therapeutic gene, which is helpful when a quick response is needed. Human Adenovirus type 5 (HAdV-C5) typically delivers DNA without inserting it into the host genome and has a low-to-no risk of insertional mutagenesis (65). However, because it does not integrate and is not replicative, AdV gene expression can be diluted and weakened over time. In addition, AdV vectors can trigger a strong immune response, which makes repeated use difficult (66). Although this is important for vaccine candidates that are typically administered repeatedly by the intramuscular route, their systemic use as a bolus for the treatment of inflammatory diseases might be less relevant. In fact, current approved gene therapies (e.g., Adstiladrin, a non-replicative HAdV-C5 expressing IFN alpha-2b) are given multiple times at 3-month intervals (67). As with other gene therapies (68, 69), high doses can generate toxicity and require monitoring. However, AdV vectors have been given to millions of humans, with far fewer serious side effects than other gene therapies and produce lethal outcomes less frequently than AAV-based gene therapies, which recent reports indicate occur at a rate of 4.7% among treated patients (70). Adenoviruses exhibit varying capacities to bind coagulation factors, creating major hurdles for both AdV gene therapy and vaccine delivery (71). These AdV-coagulation factor interactions may lead to liver sequestration, rapid vector clearance, systemic inflammation, and rare thrombotic events (72–74); however, engineered AdV vectors are being developed that are unable to bind coagulation factors (75–78). Vaccine-induced immune thrombocytopenia and thrombosis (VITT) is a rare disorder associated with the production of antibodies that react with Platelet Factor-4 (PF4) and are triggered by AdV vector vaccines and AdV infection (79). A recent study by Wang et al. reported a genetic link that contributes to VITT. In individuals carrying the immunoglobulin light-chain alleles IGLV3-21*02* or *03*, a recurrent somatic hypermutation arises that alters antibodies recognizing a defined epitope on the adenoviral core protein pVII, thereby redirecting their specificity toward PF4 (80).

In summary, our data indicate that AdV.C3-Tat/HIV-Box A exert anti-inflammatory effects in response to multiple models of local and systemic HMGB1-mediated inflammation. This study advances a first-in-class host-directed gene therapy with potential broad applications as an adjuvant to existing therapies or as a rapid-response countermeasure against emerging respiratory pathogens.

## MATERIALS AND METHODS

### Reagents

Protein-free *Escherichia coli* K235 LPS was prepared by the hot phenol-water method (81). AdV.C3-Tat/HIV-Box A and AdV.C3-Tat/HIV-Luc vectors were engineered as previously detailed (21). Final plasmids containing the genetically modified adenoviral genome were fully sequence confirmed. Viruses were purified using two rounds of CsCl ultracentrifugation and had infectious titers >10^11^ PFU/mL and physical titers >10^12^ vp/mL as previously described (21). Oseltamivir was purchased from Anta Pharma.

### Mice and CR

All work presented in this paper was conducted under strict adherence to the recommendations in the Guide for the Care and Use of Laboratory Animals of the National Institutes of Health. Six- to eight-week-old, male and female WT C57BL/6J mice were purchased from The Jackson Laboratory (Bar Harbor, ME). Six- to eight-week-old RAGE^−/−^ mice (on a C57BL/6J background; kindly provided by Dr. Ann Marie Schmidt, NYU) were bred in the fully AAALAC-accredited animal facility of the University of Maryland, Baltimore. All mouse experiments were conducted with Institutional Animal Care and Use Committee (IACUC) approval from the University of Maryland, Baltimore.

For CR studies, the animal protocols were approved by the IACUC of Sigmovir Biosystems Inc. (SBI). SBI has USDA breeding and research licenses (51-A-0031 and 51-R0091, respectively) and is AAALAC-accredited. Four- to six-week-old female and male CR (*Sigmodon hispidus*) of ~100 g were obtained from the inbred colony maintained at SBI and were housed in large polycarbonate cages and fed a diet of standard rodent chow and water *ad libitum*. CR were seronegative by ELISA for other adventitious respiratory viruses (e.g., pneumonia virus of mice, rat parvovirus, rat coronavirus, Sendai virus).

### Viruses

Mouse-adapted H1N1 influenza A/PR/8/34 virus (“PR8”) (ATCC, Manassas, VA, USA) was propagated in Madin-Darby canine kidney cells as previously described (82). RSV A2 was obtained from ATCC (Manassas, VA, USA) and propagated in Hep2 cells after serial plaque purifications to reduce defective-interfering particles. A pool of virus was designated as RSV/A2 Lot 092215 SSM containing approximately 3 × 10^9^ PFU/mL in sucrose-stabilized media and was used for the *in vivo* experiment (83).

### Mouse and CR virus challenge and treatments

For all survival experiments, mice were infected with mouse-adapted influenza virus, strain A/PR/8/34 (PR8; ~7,500 TCID_50_ i.n., 25 μL/nares), a dose of PR8 that kills ~90%–100% of infected WT mice (16, 17). Mice were treated with either saline, AdV.C3-Tat/HIV-Luc, an equal mixture of AdV.C3-Tat/HIV-Box A^Ser^ and AdV.C3-Tat/HIV-Box A^Gly^ vectors, or the individual AdV.C3-Tat/HIV-Box A^Ser^ variant at the doses and routes indicated in the experimental details provided in Results or in the figure legends. Oseltamivir (1 mg/mouse) was administered by oral gavage at the times indicated p.i. For all survival studies, mice were monitored daily for 14 days. A clinical score ranging from 0 (no symptoms) to 4 (moribund) was ascribed to each mouse daily (16). For analysis of lung or liver tissue, mice were PR8-infected on day 0 and were treated with saline (mock), AdV.C3-TAT/HIV-Luc, or AdV.C3-Tat/HIV-Box A^Ser/Gly^, and the sera collected, mice euthanized, and lungs harvested on day 5 p.i. for histopathology and gene expression analysis.

CR were infected i.n. under isoflurane anesthesia by inoculation of 100 μL (5 × 10^5^ PFU/CR) of RSV. Treatments (detailed in Results and the figure legends) were administered i.p. All CR were euthanized by carbon dioxide asphyxiation at 5 days p.i. and lungs were collected for histology and gene expression analysis as previously described (21).

### Analysis of *in vivo* responses to LPS

WT C57BL/6J mice were administered LPS (300 μg/mouse in 100 μL i.p. or 25 μg/mouse in 25 μL i.t. and treated with saline or AdV.C3-Tat/HIV-Box A after LPS as indicated. Mice challenged i.p. with LPS were monitored daily for 7 days. Mice challenged i.t. with LPS were euthanized 2 days post-treatment for lung histopathology and mRNA analysis by quantitative real-time PCR (qRT-PCR) as previously described (84).

### Cecal ligation and puncture

A clinically relevant mouse model of sepsis, CLP, was performed as described previously with minor modifications (85). Briefly, C57BL/6J male mice (10 weeks of age) were anesthetized with ketamine (100 mg/kg) and xylazine (10 mg/kg). Laparotomy was performed, and the cecum was ligated approximately 1.5 cm from the tip and punctured with a 25-gauge needle, with gentle expression of 1 mm of fecal material on both sides. The cecum was returned to the abdominal cavity. Sham-operated mice underwent laparotomy without CLP. The abdominal wall was closed with sterile sutures. After surgery, prewarmed saline (50 mL/kg body weight) was administered subcutaneously to both sham and CLP mice. Bupivacaine (3 mg/kg) and buprenorphine (0.1 mg/kg) were given pre-operatively to manage pain. AdV.C3-Tat/HIV-Luc (2 × 10^8^ PFU) or AdV.C3-Tat/HIV-Box A^Ser/Gly^ variant (2 × 10^8^ PFU) was administered i.p. 2 h after CLP surgery. Survival was monitored for 7 days in total.

### Measurement of AST and ALT as markers of liver injury

To assess vector toxicity, hepatic liver enzymes, AST and ALT, were analyzed in mice and CR. ALT and AST levels were measured by ELISA according to the manufacturer’s protocols (AFG Bioscience). C57BL/6J mice were injected (i.p.) with AdV.C3-Tat/HIV-Luc (2 × 10^8^ PFU/mouse or 2 × 10^9^ PFU/mouse). Serum was collected after 24 h. CR were pre-bled (0 h) and then administered PBS or AdV.C3-Tat/HIV-Box A^Ser^ i.p. (1 × 10^8^ or 1 × 10^9^ PFU). Serum was collected 2 and 24 h post-AdV.C3-Tat/HIV-Box A^Ser^ administration. CR were then injected with LPS (1 mg/kg; i.p.) and serum collected 24 h post-LPS.

For APAP studies, C57BL/6J male mice were fasted overnight and received APAP (350 mg/kg) by i.p. injection (50). On days 1 and 3 post-APAP, mice were administered AdV.C3-Tat/HIV-Luc (2 × 10^8^ PFU/mouse) or AdV.C3-Tat/HIV-Box A^Ser^ (2 × 10^8^ PFU/mouse) i.p. Some groups of mice were monitored for survival for 14 days post-APAP, while other groups of mice were used for harvest studies. For the harvest studies, on day 5 post-APAP, serum was collected for liver enzyme analysis by ELISA. Mice were then euthanized, and the livers harvested for histology and gene expression analysis.

### Analysis of *in vitro* responses to LPS

Human HL-60 cells (ATCC) were cultured in RPMI 1640 growth medium that was supplemented with fetal bovine serum (10%), penicillin-streptomycin (10 U/mL), and L-glutamine (2 mM). Prior to experimentation, HL-60 cells were differentiated into adherent Mϕ-like cells by overnight treatment with PMA (100 nM) in medium containing 2% fetal bovine serum. Non-adherent cells were aspirated, and fresh growth medium containing 2% fetal bovine serum was added. For experimentation, cells were treated with media alone or infected with AdV.C3-Tat/HIV-Box A^Gly^ (MOI = 2) for 24 h, then treated with media alone or LPS (10 ng/mL) for 18 h. Gene expression was analyzed by qRT-PCR.

### Quantitative real-time PCR

qRT-PCR was performed on total RNA using an ABI Prism QuantStudio 3 Sequence Detection System with SYBR Green reagent (Applied Biosystems) and transcript-specific primers (21, 86, 87). Levels of mRNA for specific genes are reported as relative gene expression normalized to that of WT mock-infected lungs, livers, or Mϕ (“fold induction”) (88). The housekeeping gene, hypoxanthine-guanine phosphoribosyltransferase (*Hprt*), was used for normalization of RNA levels. The following primer set was used for detection of HAdVpan: forward 5′-GCCCCAGTGGTCTTACATGCACATC-3′ and reverse 5′-GCCACGGTGGGGTTTCTAAACTT-3′ (89).

### Histology and staining

Lungs were inflated, perfused, and fixed with 4% PFA. Fixed sections (5 mm) of paraffin-embedded lungs were stained with hematoxylin and eosin (H&E). Four inflammatory parameters were scored independently from 0 to 4 for each section: peribronchiolitis (inflammatory cells, primarily lymphocytes, surrounding a bronchiole), perivasculitis (inflammatory cells, primarily lymphocytes, surrounding a blood vessel), alveolitis (inflammatory cells within alveolar spaces), and interstitial pneumonitis (increased thickness of alveolar walls associated with inflammatory cells). Slides were randomized, read blindly, and scored for each parameter. Individual pathology scores for each parameter were then converted to percentage damage for each parameter following a fixed rule where 0 = 0, 1 = 5%, 2 = 25%, 3 = 75%, and 4 = 100% (90).

### Statistics

Statistical differences between two groups were determined using an unpaired, one-tailed Student’s *t* test with significance set at *P* < 0.05. For comparisons between ≥3 groups, analysis was done by one-way ANOVA, followed by a Tukey’s multiple comparison post hoc test with significance determined at *P* < 0.05. For survival studies, a Mantel-Cox log-rank test was used.

## AFTER EPUB

*[This article was published on 24 August 2026 with an error in the Direct Contribution footnote. The footnote corrected in the current version, posted on 25 August 2026.]*

## SUPPLEMENTAL MATERIAL

Fig. S1 mbio.01819-26-s0001.pdf Effect of AdV vectors on liver enzymes.

ASM does not own the copyrights to Supplemental Material that may be linked to, or accessed through, an article. The authors have granted ASM a non-exclusive, world-wide license to publish the Supplemental Material files. Please contact the corresponding author directly for reuse.
