# Supplementary material for: HMGB1-targeted anti-inflammatory rBox A gene therapy for viral airway infections and systemic inflammatory disease models
Source: mBio. 2026 Aug 24;17(10):e01819-26. doi: 10.1128/mbio.01819-26 (PMC13712864; doi:10.1128/mbio.01819-26)
Supplement: Supplementary file 1 [file mbio.01819-26-s0001.pdf]

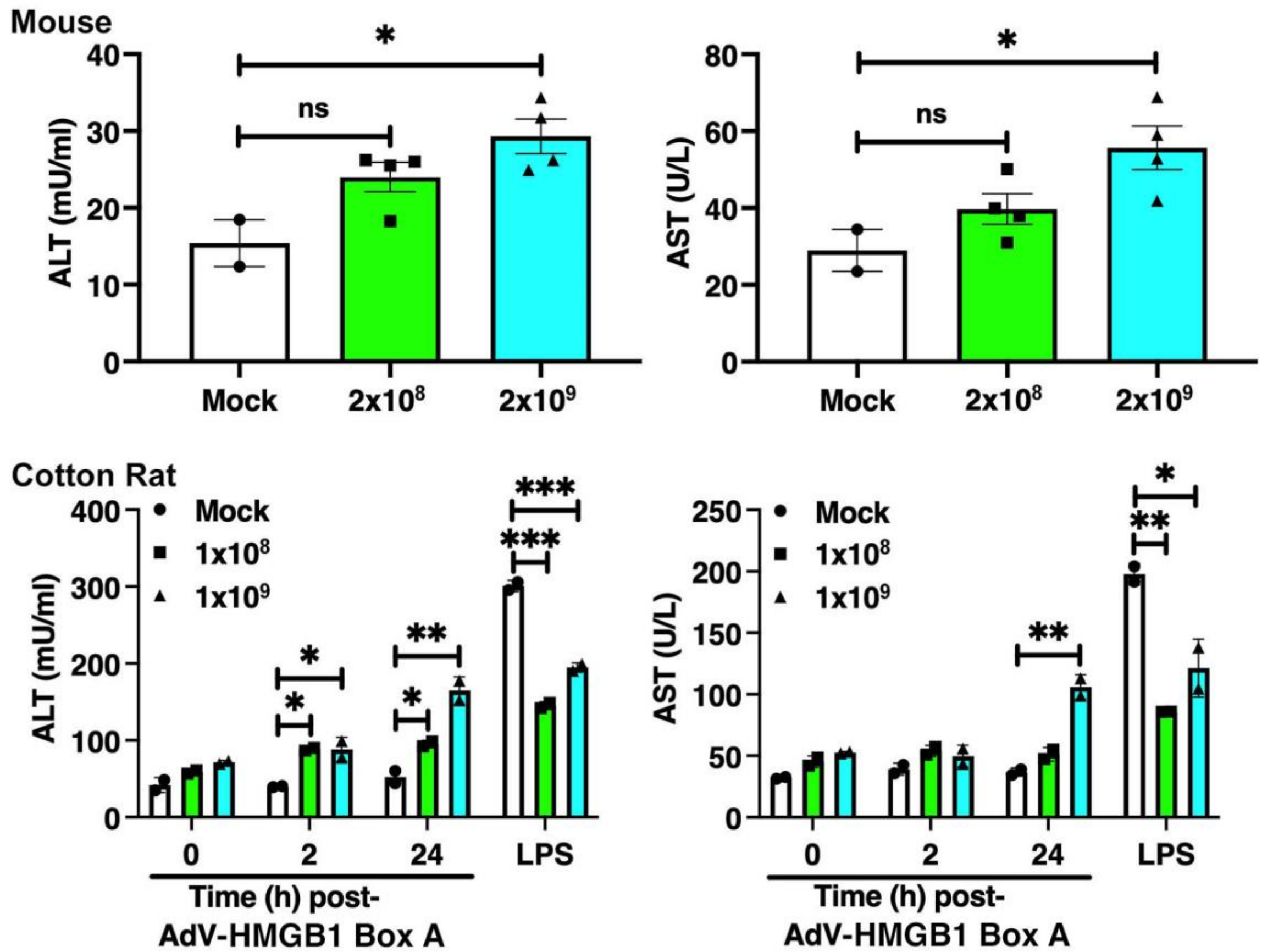

Supplemental Figure 1. Effect of AdV vectors on liver enzymes. (A). C57BL/6J mice were injected i.p. with saline or increasing concentrations of AdV.C3-Tat/HIV-Luc ($2x10^8$ or $2x10^9$ PFU/mouse). After 24 h, serum was collected and AST and ALT levels measured by ELISA. (B) CR were pre-bled (0 h) and then injected i.p. with PBS or AdV.C3-Tat/HIV-Box A^{Ser} ("AdV-HMGB1 Box A"; $1x10^8$ PFU or $1x10^9$ PFU/CR) and serum collected 2 and 24 h later. All CR were then injected i.p. with LPS (dose) and serum collected 24 h later for liver enzyme measurements by ELISA. N = 3 CR/group. Each symbol represents one mouse or CR.
